# Supplementary material for: Nonstimulatory peptide–MHC enhances human T-cell antigen-specific responses by amplifying proximal TCR signaling
Source: Nat Commun. 2018 Jul 13;9:2716. doi: 10.1038/s41467-018-05288-0 (PMC6045629; doi:10.1038/s41467-018-05288-0)
Supplement: Supplementary file 1 — Supplementary Information [file 41467_2018_5288_MOESM1_ESM.pdf]

Non-stimulatory peptide-MHC enhances human T cell antigen-specific responses by amplifying proximal TCR signaling

Zhao *et al.*

Supplementary Information

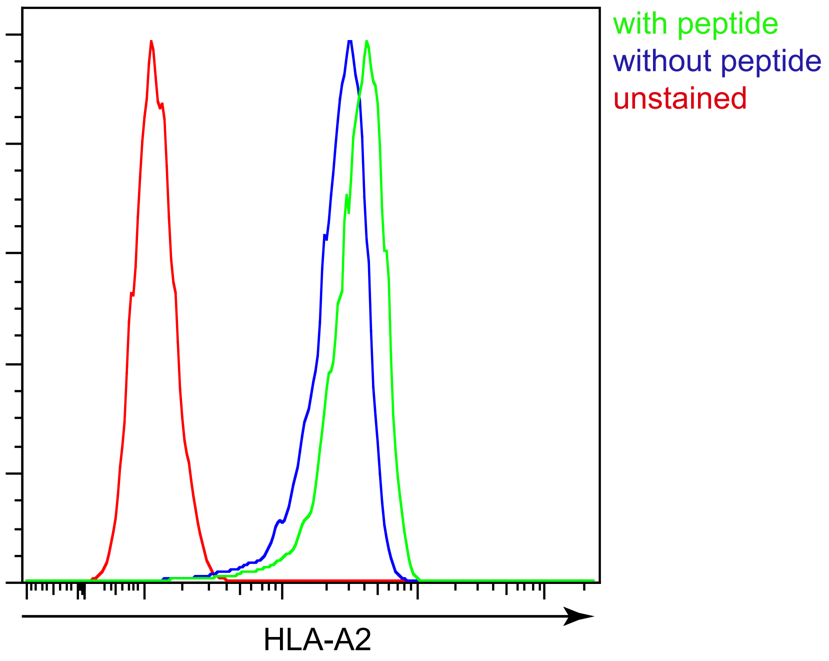

Supplementary Fig. 1. T2 cell line expresses high amount of pMHC on the surface. T2 cells pulsed with or without 1 μM HLA-A*0201-restricted peptides E183 (FLLTRILTI) for 1h were stained with anti-HLA-A2 antibody and analyzed by flow cytometry.

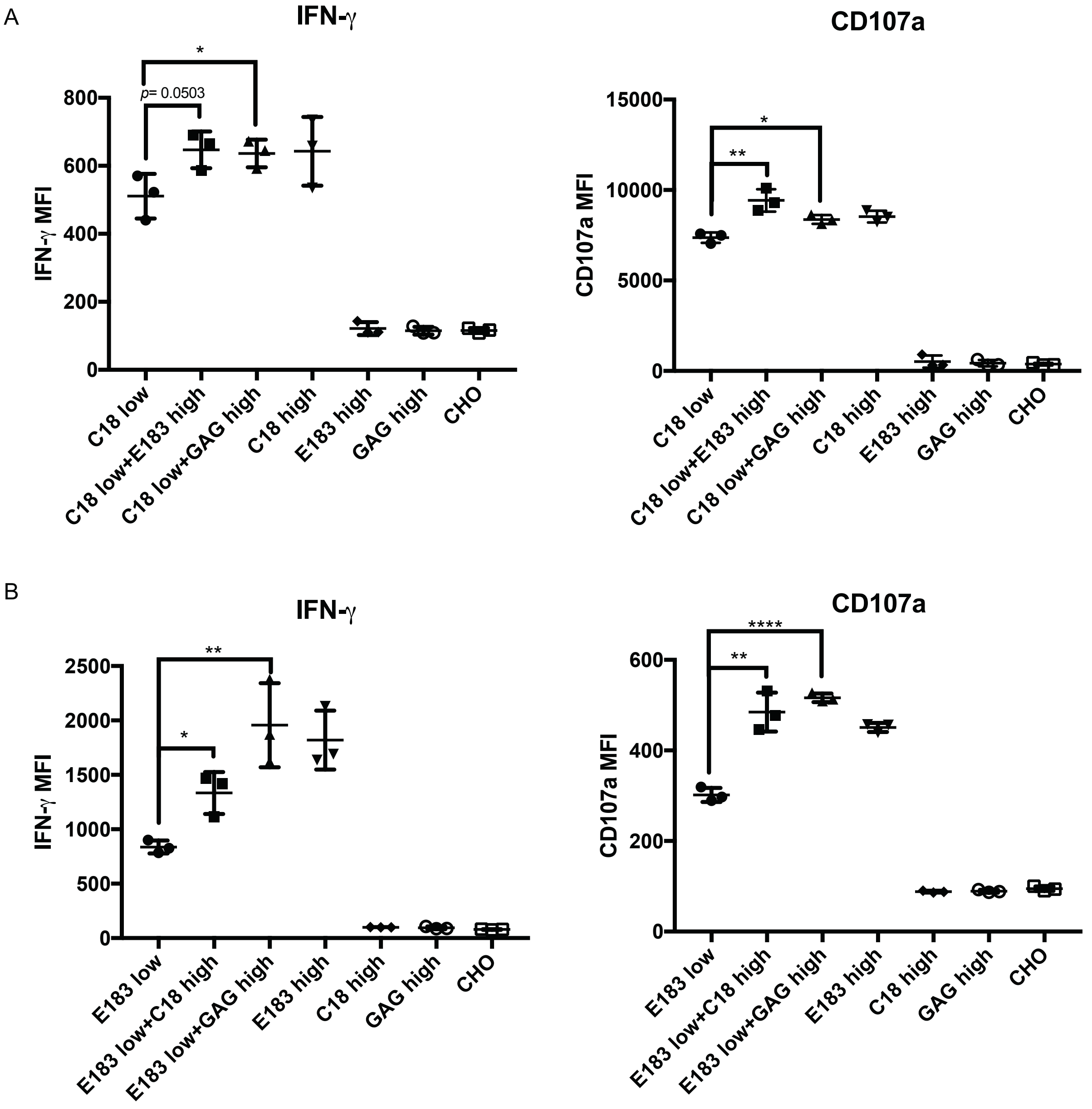

Supplementary Fig. 2. Non-stimulatory pMHC enhance human TCR-Td T cells response to antigen. C18-specific-TCR-Td T cells (A) or E183-specific-TCR-Td T cells (B) were cocultured with indicated T-REx CHO cells panel for 3 h, and the production of IFN-γ and upregulation of the degranulation marker CD107a were assessed by flow cytometry. Statistical significance was determined by using unpaired two-sided Student's t-test and shown as mean ± s.d. (*, p<0.05, **, p< 0.01, ***, p<0.001). Data are representative of three independent experiments.

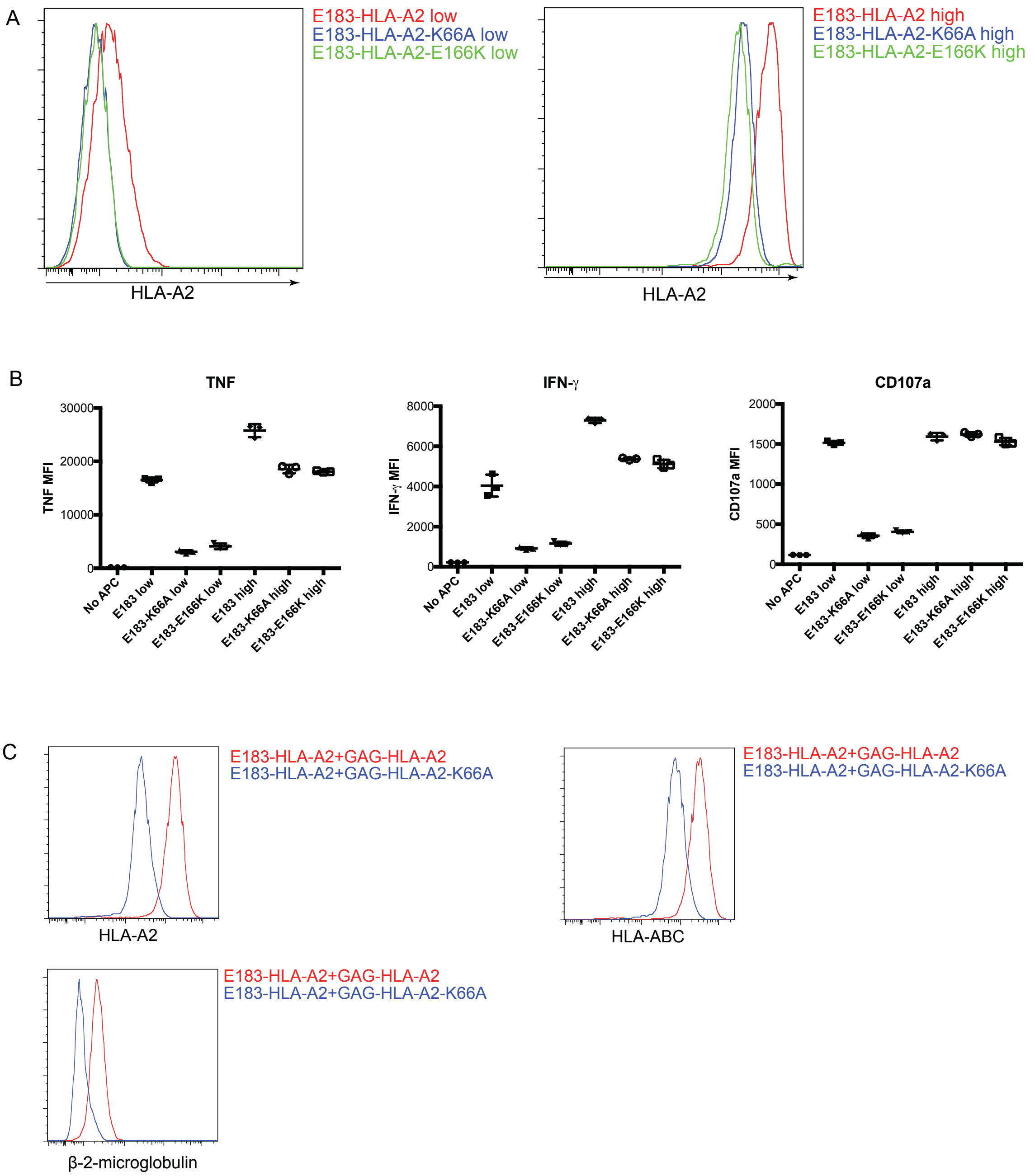

Supplementary Figure 3. K66A and E166K mutations in HLA-A2 heavy chain do not abrogate TCR binding to HLA-A2. (A) K66A or E166K mutation was introduced in the antigen sc E183-HLA-A2. Doxycycline-inducible sc E183-HLA-A2-K66A or sc E183-HLA-A2-E166K constructs were transfected into T-REx CHO cells and single cell clones were selected. Low or high expression (doxycycline-induced) was detected by anti-HLA-A2 staining and flow cytometry. (B) Human E183 CTL were cultured with the indicated T-REx CHO cells for 3 h and cytokine production (TNF and IFN-γ) and upregulation of the degranulation marker CD107a were assessed by flow cytometry. Data are representative of three independent experiments. (C) Anti-HLA-A2, anti-HLA-ABC, anti-β-2-microglobulin staining of T-REx CHO clones expressing low amount of sc E183-A2 with either high expression of sc GAG-A2 or high expression of GAG-A2-K66A.

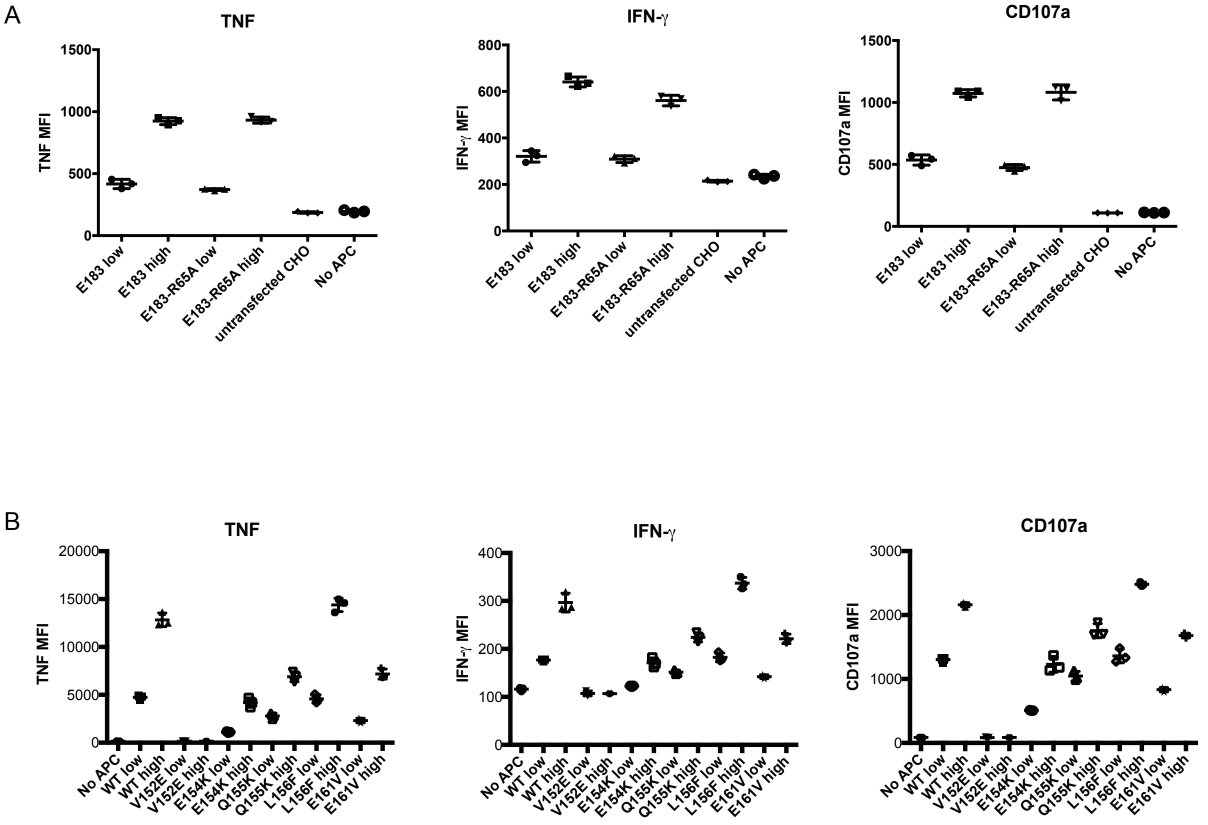

Supplementary Figure 4. V152E mutation on HLA-A2 heavy chain can abolish TCR recognition of peptide-HLA-A2. (A) Human E183 CTL were cultured with the indicated T-REx CHO cells for 3 h and cytokine production (TNF and IFN-γ) and upregulation of the degranulation marker CD107a were assessed by flow cytometry. (B) Human E183 CTL were cultured with indicated T-REx CHO cells for 3 h and cytokine production (TNF and IFN-γ) and upregulation of the degranulation marker CD107a were assessed by flow cytometry.

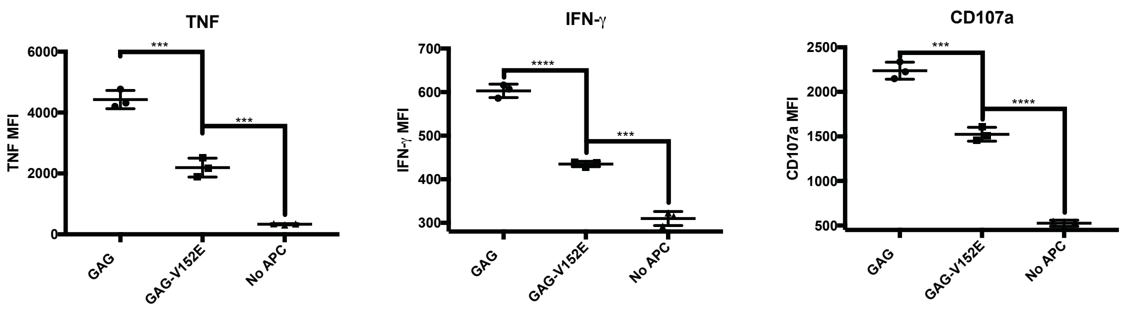

Supplementary Figure 5. V152E mutation abrogates specific TCR recognition of GAG-A2 complex. GAG-A2-specific T cells were cocultured with the indicated CHO cells panel for 3 h, and the production of cytokine (TNF and IFN-γ) and upregulation of CD107a were measured by flow cytometry. Statistical significance was determined by using unpaired two-sided Student's t-test and shown as mean ± s.d. (*, p<0.05, **, p< 0.01, ***, p<0.001, ****, p<0.0001). Representative of 2 experiments.

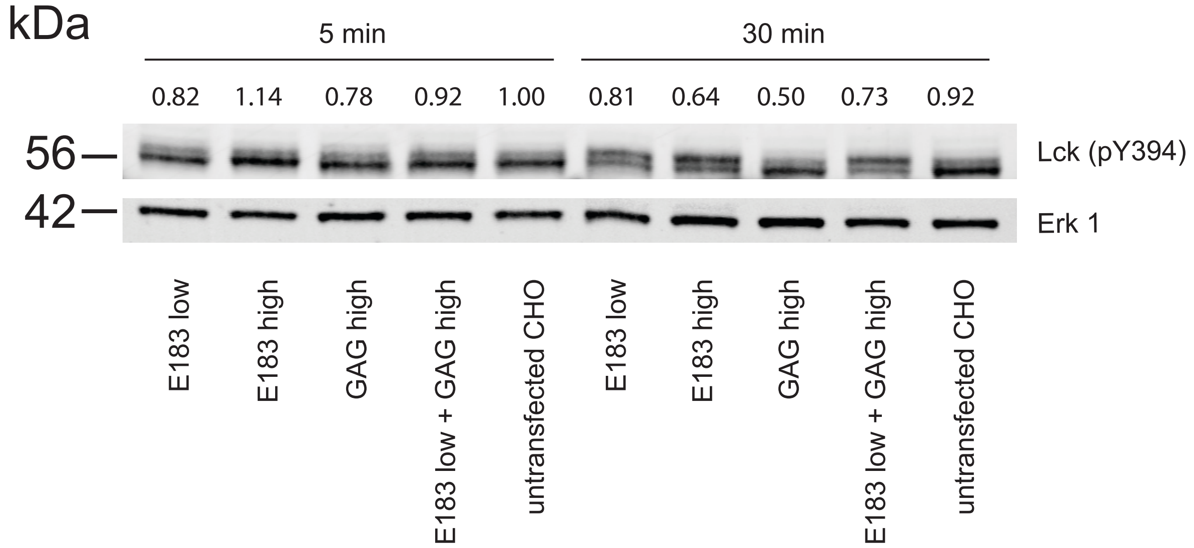

Supplementary Fig. 6. The amount of active Lck was not changed during coagonism. Human E183 CTL were stimulated by the indicated CHO cell panel for 5 min or 30 min, lysed and then separated on NuPAGE Bis-Tris Gel. pLck were probed and analysed on the LI-COR Odyssey infrared imaging system. Anti-pSrc (Y416) antibody was used to detect active Lck (pY394). The data are representative of three independent experiments. Uncropped western blotting imaging is presented in Supplementary Fig. 8.

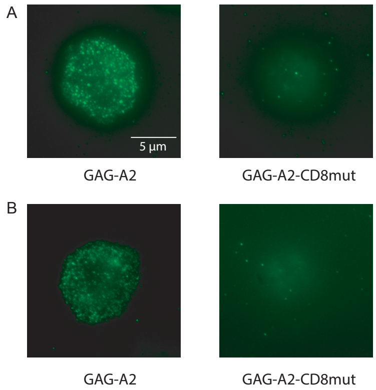

Supplementary Fig. 7. The formation of the immunological synapse with non-stimulatory pMHC monomer requires intact CD8 binding to the pMHC. 1 µM GAG-A2 or GAG-A2-CD8mut monomer was added to the lipid bilayers in a glass chamber slide. 10^5 E183 CTL were added into each well of the chamber and stimulated for 5 min. The CTL were fixed, permeabilized and stained with pLck (A), or pZap70 (B) antibody. The formation of the immunological synapse was observed by TIRF microscopy. Anti-pSrc (Y416) antibody was used to detect active Lck (pY394).

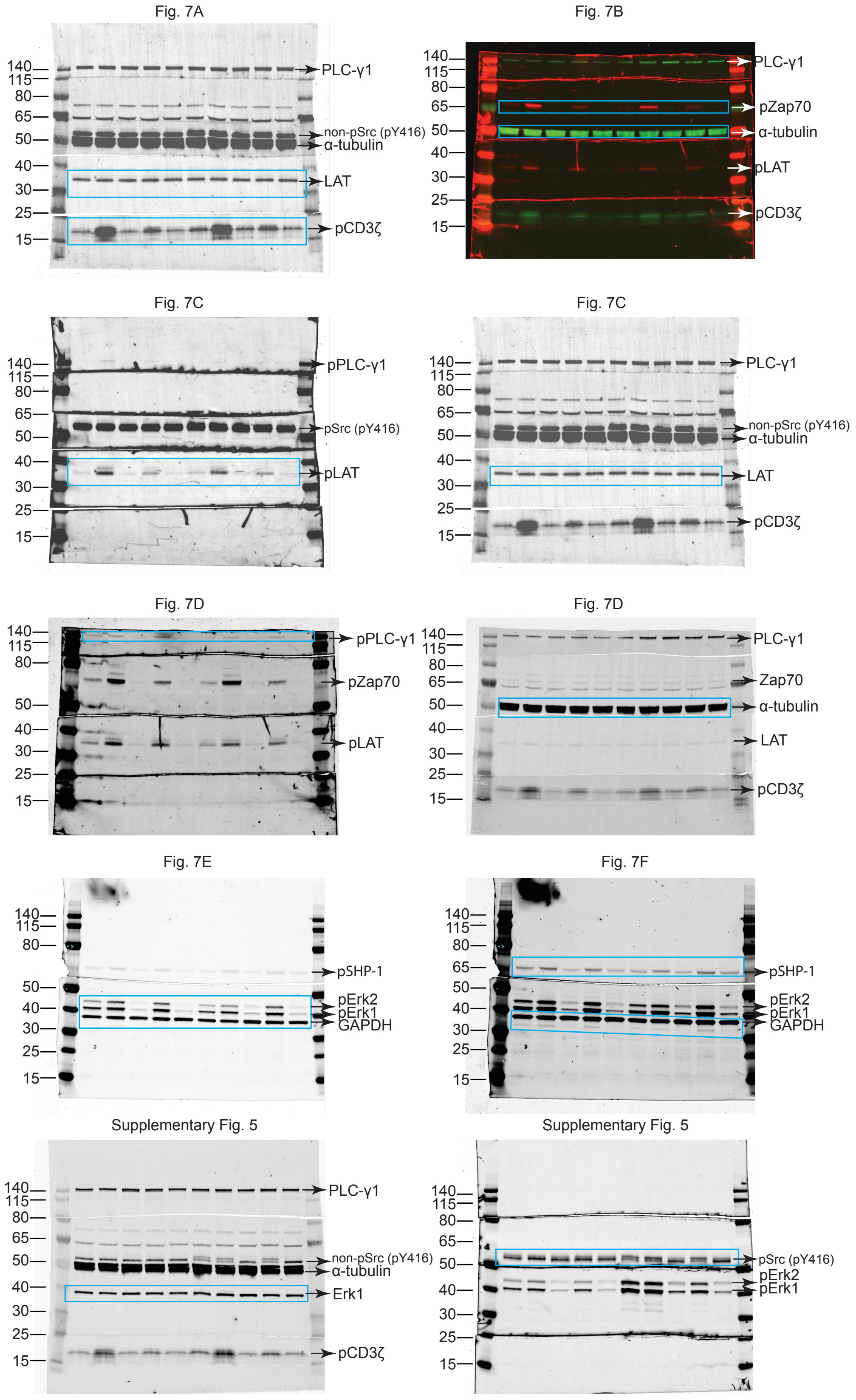

Supplementary Fig. 8. Uncropped western blotting images related to Fig. 7 and Supplementary Fig. 6. Each membrane was cut into several pieces and each piece was blotted with different primary antibodies.

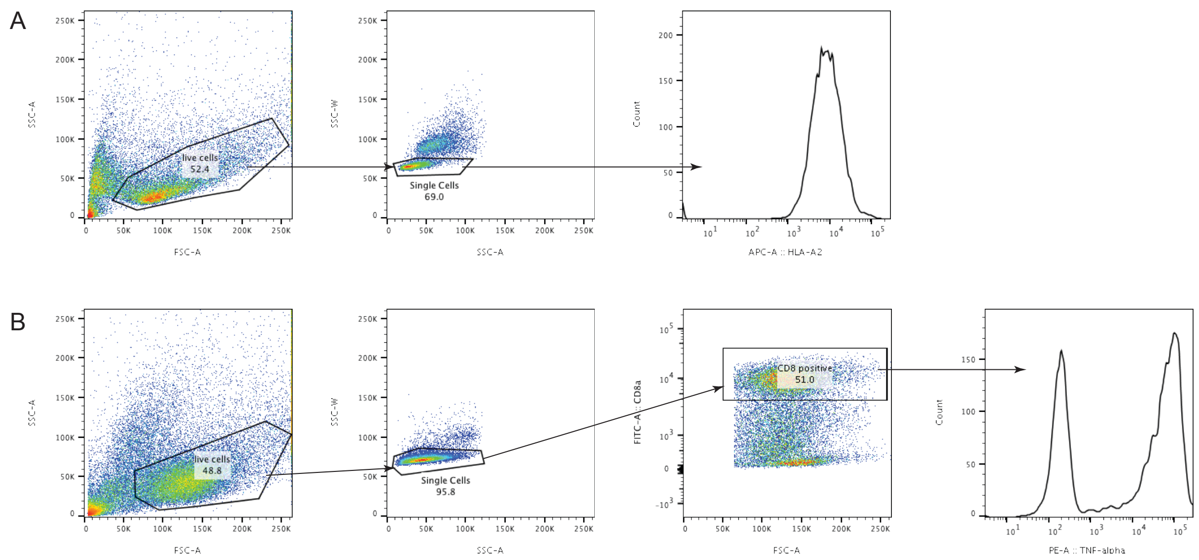

Supplementary Fig.9. Gating strategies used in this project. (A) Gating strategy to measure MHC expression on the surface of CHO cells in Fig. 1A. The same strategy was used for Fig. 1A-C, Fig. 2A, Fig. 3B, Fig. 4B-C, Supplementary Fig. 1, Supplementary Fig. 3A, C. (B) Gating strategy to measure the production of cytokine, and upregulation of degranulation marker CD107a in human E183 CTL in Fig. 1d. The same strategy was used for Fig. 2C, Fig. 3A, D, Fig. 4A, D, Fig. 5, Fig. 6, Supplementary Fig. 2, Supplementary Fig. 3, Supplementary Fig. 4.

Supplementary Note 1

All the primers were ordered from IDT, Inc.

Single chain trimer of GAG-A2 was cloned into pcDNA5TO vector. The primers were:

Forward: 5’-CAGCAGAAGCTTCAGCATGTCTCGCTCCGTGGCCTT-3’,

Reverse: 5’-CAGCAGCTCGAGTCACACTTTACAAGCTGTGA-3’

In GAG-A2-pcDNA5TO recombinant construct, the GAG peptide was mutated into E183-91 peptide by two-round mutagenesis. The following primers were used for the first-round mutagenesis:

Forward: 5'-TTTCTGGCCTGGAGGCTTTCCTGCTCACCACCGTCGCGACCCTAG-3'

Reverse: 5'-CTAGGGTCGCGACGGTGGTGAGCAGGAAAGCCTCCAGGCCAGAAA-3'

The following primers were used for the second-round mutagenesis:

Forward: 5'-GCCTCCCCCACCTCCTATGGTCAGGATGCGGGTGAGCAGGAAAGCCT-3'

Reverse: 5'-AGGCTTTCCTGCTCACCCGCATCCTGACCATAGGAGGTGGGGGAGGC-3'

In E183-91-A2-pcDNA5TO recombinant construct, the E183-91 peptide was mutated into C18-27 peptide by two-round mutagenesis. The following primers were used for the first-round mutagenesis:

Forward: 5'-CCCCACCTCCTATGGTCAAGAAGTCGGAGGGCAGGAAAGCCTCCAGGC-3'

Reverse: 5'-GCCTGGAGGCTTTCCTGCCCTCCGACTTCTTGACCATAGGAGGTGGGG-3'

The following primers were used for the second-round mutagenesis:

Forward: 5'-CCTCCCCCACCTCCGACGCTGGGGAAGAAGTCGGAGGGCAGG-3'

Second: 5'-CCTGCCCTCCGACTTCTTCCCCAGCGTCGGAGGTGGGGGAGG-3'

The single chain trimer of GAG-A2, E183-91-A2, and C18-27-A2 were cloned into pcDNA3-Clover vector by using the following primers:

Forward: 5’- CAGCAGAAGCTTCAGCATGTCTCGCTCCGTGGCCTT-3’

Reverse: 5’- CAGCAGGGATCCCACTTTACAAGCTGTGAGAG-3'

For the D227K, T228A mutagenesis in the heavy chain of A2, the following primers were used:

Forward: 5'-TCTCCACGAGCTCCGCCTTCTGGGTCTGGTCCTC-3'

Reverse: 5'-GAGGACCAGACCCAGAAGGCGGAGCTCGTGGAGA-3'

For the A245V mutagenesis in the heavy chain of A2, the following primers were used:

Forward: 5'-GGAACCTTCCAGAAGTGGGTGGCTGTGGTGGTGCCTTCTG-3'

Reverse: 5'-CAGAAGGCACCACCACAGCCACCCACTTCTGGAAGGTTCC-3'

For the Q115E mutagenesis in the heavy chain of A2, the following primers were used:

Forward: 5'-CGTAGGCGTACTCGTGGTACCCGCG-3'

Reverse: 5'-CGCGGGTACCACGAGTACGCCTACG-3'

For the K66A mutagenesis in the heavy chain of A2, the following primers were used:

Forward: 5'-GAGTGGGCCTTCACTGCCCGTGTCTCCCCGTC-3'

Reverse: 5'-GACGGGGAGACACGGGCAGTGAAGGCCCACTC-3'

For the R65A mutagenesis in the heavy chain of A2, the following primers were used:

Forward: 5'-GGGCCTTCACTTTCGCTGTCTCCCCGTCCC-3'

Reverse: 5'-GGGACGGGGAGACAGCGAAAGTGAAGGCCC-3'

For the E166K mutagenesis in the heavy chain of A2, the following primers were used:

Forward: 5'-TGCGGAGCCACTTCACGCACGTGCC-3'

Reverse: 5'-GGCACGTGCGTGAAGTGGCTCCGCA-3'

For the V152E mutagenesis in the heavy chain of A2, the following primers were used:

Forward: 5'-ACTGCTCCGCCTCATGGGCCGCC-3'

Reverse: 5'-GGCGGCCCATGAGGCGGAGCAGT-3'

For the E154K mutagenesis in the heavy chain of A2, the following primers were used:

Forward: 5'-GCTCTCAACTGCTTCGCCACATGGGCC-3'

Reverse: 5'-GGCCCATGTGGCGAAGCAGTTGAGAGC-3'

For the Q155K mutagenesis in the heavy chain of A2, the following primers were used:

Forward: 5'-GTAGGCTCTCAACTTCTCCGCCACATGGG-3'

Reverse: 5'-CCCATGTGGCGGAGAAGTTGAGAGCCTAC-3'

For the Q155A mutagenesis in the heavy chain of A2, the following primers were used:

Forward: 5'-GGTAGGCTCTCAACGCCTCCGCCACATGGG-3'

Reverse: 5'-CCCATGTGGCGGAGGCGTTGAGAGCCTACC-3'

For the L156F mutagenesis in the heavy chain of A2, the following primers were used:

Forward: 5'-CAGGTAGGCTCTGAACTGCTCCGCCAC-3'

Reverse: 5'-GTGGCGGAGCAGTTCAGAGCCTACCTG-3'

For the E161V mutagenesis in the heavy chain of A2, the following primers were used:

Forward: 5'-ACGCACGTGCCCACCAGGTAGGCTC-3'

Reverse: 5'-GAGCCTACCTGGTGGGCACGTGCGT-3'
